# Radiochemotherapy and interstitial brachytherapy for cervical cancer: clinical results and patient-reported outcome measures

**DOI:** 10.1007/s00066-023-02196-1

**Published:** 2024-01-31

**Authors:** Johanna Alfrink, Thomas Aigner, Hermann Zoche, Luitpold Distel, Gerhard G. Grabenbauer

**Affiliations:** 1 Department of Radiation Oncology, University Hospitals of Erlangen, Erlangen, Germany; 2 Department of Radiation Oncology, Coburg Cancer Center, Coburg, Germany; 3 Department of Gynecology and Obstetrics, Coburg Cancer Center, Coburg, Germany; 4 Department of Pathology, Coburg Cancer Center, Coburg, Germany; 5 Department of Radiation Oncology, Coburg Cancer Center, Coburg, Germany

**Keywords:** Radiochemotherapy, Locally advanced cervical cancer, External radiation and interstitial brachytherapy, Quality of life, Questionnaires

## Abstract

**Objective:**

To evaluate clinical results and long-term patient-reported outcome measures (PROMs) on quality of life in cervical cancer patients following radiochemotherapy (RCT) and brachytherapy (BT) as definitive treatment.

**Materials and methods:**

Between 2003 and 2023, a total of 132 patients with advanced cervical cancer were evaluated for possible treatment. Patients treated by postoperative RCT, palliative radiotherapy, and those treated for recurrent disease were excluded. Thus, 46 patients receiving standard RCT and BT as their curative treatment were included in this study. PROMs were assessed prospectively by patients’ self-completion of the EORTC-QLQ-C30 and EORTC-QLQ-CX24 questionnaires.

**Results:**

Five-year overall survival (OS), distant metastases-free survival (DMFS), and pelvic tumor-free survival rates (PTFS) were 53%, 54%, and 83%, respectively. A significant impact on OS was seen for FIGO (International Federation of Gynecologic Oncology) stage (IIB–IIIA: 79% vs. IIIB–IVA: 33%, *p* = 0.015), for overall treatment time (OTT; 50–65 d: 64% vs. > 65 d: 38%, *p* = 0.004), and for rectal D_2cc_ (≤ 73 Gy: 50% vs. > 73 Gy: 38%, *p* = 0.046). The identical parameters were significantly associated with DMFS (FIGO stage: *p* = 0.012, OTT: *p* = 0.008, D_2cc_: *p* = 0.024). No parameters with a significant influence on PTFS were seen. In multivariate analysis, an impact of FIGO stage on OS (*p* = 0.05) and DMFS (*p* = 0.014) was detected, and of rectal D_2cc_ on DMFS (*p* = 0.031). The overall QoL score was 63/100. Cognitive function was the least impaired (84/100), while role functioning was the worst (67/100). On the symptom scale, insomnia (46/100), fatigue (41/100), dyspnea (32/100), pain (26/100), and financial difficulties (25/100) were scored the worst. According to EORTC-QLQ-CX24, peripheral neuropathy (36/100) and lymphedema (32/100) occurred most frequently. Impaired sexual/vaginal functioning (32/100) and body image (22/100) were also frequently recorded.

**Conclusion:**

In patients with advanced cervical cancer, a combination of RCT and BT remains an excellent treatment option. In terms of patient-reported long-term quality of life, specific support is needed to alleviate symptoms including lymphedema, peripheral neuropathy, and impaired sexual activity.

## Introduction

Cervical cancer ranks as the second most prevalent gynecological malignancy and the fourth most common cancer among women [1]. Treatment options vary based on tumor stage and have changed significantly over the past few years. To ensure the most effective treatment plan for each patient, a multidisciplinary tumor board consultation is necessary [2]. According to the FIGO (International Federation of Gynecologic Oncology) guidelines, surgical treatment is confined to early-stage disease. For locally advanced cervical cancer, namely FIGO stages IIB to IVA, treatment using radiochemotherapy followed by brachytherapy is standard [3]. Cancer and its treatment may have a significant impact on patients in terms of morbidity, mortality, and QoL. In addition to coping with the immediate side effects of cancer therapy, physical, social, and psychological/emotional functioning are evenly affected and should also be taken into account. This can be measured by assessing the QoL of patients, which comprises a complex and wide-ranging issue reflecting patients’ experiences with the disease, its treatment, and the long-term consequences associated with it [4]. To properly assess QoL in cervical cancer patients, there are two official questionnaires designed by the European Organization for Research and Treatment of Cancer (EORTC). The EORTC-QLQ-C30 is a comprehensive questionnaire applicable to a wide range of cancer types, while the EORTC-QLQ-CX24 was specifically designed to address the QoL of cervical cancer patients [1]. Clinical results as well as long-term patient-reported outcome measures (PROM) on quality of life are given in this paper.

## Patients and methods

This retrospective study evaluated patient and treatment data as well as long-term results among patients treated by a combination of radiochemotherapy and interstitial brachytherapy for cervical cancer at a single institution. In addition, for the group of surviving patients, quality of life data were collected prospectively using the questionnaires EORTC QLQ-C30 (version 3) and EORTC QLQ-CX24. Ethical approval was obtained from the Ethics Committee of the University Hospitals of Erlangen (no. 23-5-B).

**Fig. 1:**
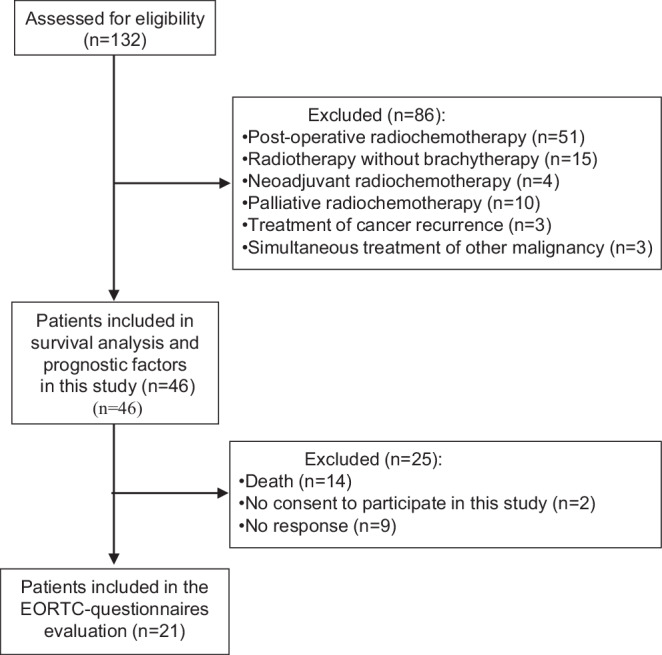
Consolidated Standards of Reporting Trials (CONSORT) flow diagram

Between 2003 and 2023, a total of 132 patients with cervical cancer were evaluated for possible treatment at this cancer center. A subgroup of 46 patients received radiochemotherapy and brachytherapy as their definitive treatment and served as the study group. Eighty-six patients were excluded for various reasons (Fig. 1). Of the remaining 46 eligible patients, 25 did not participate in the evaluation of EORTC-QLQ-C30 and EORTC-QLQ-CX24 questionnaires due to death, missing consent, or no response after receiving the questionnaires by mail. The questionnaires were sent to 32 patients in March 2023. All returns up to 6 weeks after receipt of the questionnaires by postal mail were taken into account. Before the questionnaires were sent out, the patients were contacted by telephone about participating in the study and completing the questionnaires.

### Statistical analysis

IBM SPSS Statistics 29 for macOS (IBM Corp, Armonk, NY, USA) was used to analyze the results. The Kolmogorov–Smirnov test was used to analyze the normality of the data distribution. Survival rates were calculated by the Kaplan–Meier method, and the log-rank test was used to compare survival rates between different groups of patients. Cox regression analysis was used to test for variables with an independent influence on survival parameters. Qualitative data are expressed as whole numbers and percentages, while quantitative data are expressed as mean ± standard deviation (SD) or mean and interquartile range (IQR). Results are presented as tables and figures with a 95% confidence interval (CI).

For analysis of the EORTC questionnaires, all data are summarized in descriptive statistics. To compare differences between two groups, the *t*-test was performed. The chi-square test was applied to compare two categorical variables, and regression analysis was used to examine the influence of certain variables on QoL. Frequencies are reported for categorical variables. The significance level of all tests was set at 0.05.

## Results

### Patients

Patient characteristics are given in Table 1. Mean age at initial diagnosis was 58 years (range 27–86 years). In addition to pelvic examination, all patients were staged by a CT scan of the chest and abdomen, pelvic MRI, and had biopsy-proven cervical cancer. In selected cases (21; 46%) laparoscopic paraaortic lymph node sampling was performed. FIGO stage IIA–IIIA was assigned to 24 (52%) patients, while 22 patients were classified as having FIGO stage IIIB–IVA. Squamous cell carcinoma with 39 patients (85%) was much more common than adenocarcinoma with 7 patients (15%).

**Table 1:** Patient and treatment characteristics

Variable		
Total number of patients	–	46 (100%)
FIGO stage	IIB–IIIA	24 (52%)
	IIIB–IVA	22 (48%)
Tobacco smoking	Yes	16 (35%)
	No	30 (65%)
Histology	Squamous cell carcinoma	39 (85%)
	Adenocarcinoma	7 (15%)
Menstrual status	Premenopausal	14 (30%)
	Perimenopausal	6 (13%)
	Postmenopausal	26 (57%)
Age at diagnosis	Mean	58 years
	Minimum	27 years
	Maximum	86 years
Total dose of EBRT	Mean	50.7 Gy
	IQR	49.1–50.4 Gy
Total dose of BT	Mean	25.8 Gy
	IQR	27–28 Gy
Dose paraaortic^a^ LN	Mean	13.9 Gy
	IQR	0–45.4 Gy
Dose pelvic LN	Mean	49.8 Gy
	IQR	47.3–50.4 Gy
Dose primary tumor	Mean	53.9 Gy
	IQR	50.4–56 Gy
Duration of EBRT and BT	Mean	65.4 days
	IQR	54.5–79 days
Total dose of cisplatin^b^	Mean	147.8 mg/m^2^ BSA
	IQR	40–200 mg/m^2^ BSA
Total dose at D_2cc_ bladder	Mean	77.2 Gy
	IQR	72.7–81.2 Gy
Total dose at D_2cc_ rectum	Mean	72.9 Gy
	IQR	70.8–76.8 Gy

*EBRT* external beam radiation therapy, *BT* brachytherapy, *LN* lymph nodes, *D*_*2cc*_ dose received at rectum at D = 2 cm^3^, *IQR* interquartile range, *BSA *body surface area

^a^Of the 51 patients, only 15 received paraaortic radiation

^b^Eight patients did not receive cisplatin but carboplatin or 5‑FU due to hematotoxic or nephrotoxic complications

### Treatment parameters

Following computed tomography of the abdomen and pelvis after rectal and bladder preparation (all organs empty) using spiral CT with 3 mm reconstruction (Canon, Aquilion LB©, Neuss, Germany) and MRI of the pelvis (T2w SE, T1w-Gd enhanced data, Siemens, Altea© 1.5T or Amira© 1.5T), treatment planning was performed (Varian, Eclipse©: treatment planning, v. 15.5) and applied by IMRT using dynamic arcs (Varian, TrueBeam© v. 2.7). Dose was prescribed with single fractions of 1.8 Gy and a total dose of 50.4 Gy, specified to the D50 according to ICRU report 83. Accordingly, the median total dose was 50.4 Gy (IQR: 49.1–50.4 Gy). The median dose delivered to the primary tumor volume was 54.6 Gy (IQR: 50.4–56 Gy), for the pelvic/paraaortic lymph nodes it was 50.4 Gy (IQR 47.3–50.4 Gy). Thirteen of 46 patients received radiation to the paraaortic lymph node region given positive histologic laparoscopic sampling there.

For brachytherapy, a total dose of 28 Gy in 4 fractions was administered twice weekly (IQR: 27–28 Gy) according to the technique described by Pötter et al. [5]. After insertion of the Vienna applicator (Varian, 3D Interstitial Ring Applicator, GammaMed Plus iX©) under general and peridural anesthesia, treatment planning with CT and MRI was performed. Gross tumor volume, high-risk clinical target volume, and low-risk clinical target volume were delineated according to Pötter et al. [5]. Brachytherapy dose was intended to cover the high-risk clinical target volume and was specified to the 85% isodose. Dose constraint for organs at risk were as follows: for rectum and bladder, D_2cc_ should not exceed a fractional dose of 3 Gy. Treatment characteristics are given in Table 1. The median D_2cc_ for rectum was 73.8 Gy, while the bladder received a slightly higher dose with a median of 78.3 Gy.

Patients were scheduled for intravenous cisplatin chemotherapy following a sufficient urinary excretory function test with a creatinine clearance of at least 70 mL/min. A weekly dose of 40 mg/m^2^ was to be applied. For simultaneous cisplatin, a median total dose of 200 mg/m^2^ body surface area (BSA) was applied (IQR: 40–200 mg/m^2^ BSA). A total of 5 patients had another chemotherapeutic regimen such as carboplatin or mitomycin C together with 5‑fluoruracil (5-FU) instead of cisplatin due to hematological or nephrological limitations.

### Toxic effects

According to the medical records, a total of 27 patients (57%) complained of acute toxic effects during and after treatment and 10 (22%) of the patients complained of late toxicities. Table 2 gives an overview according to the CTC grading. In general, most symptoms could be treated in an outpatient setting and were thus staged grade 2. Pulmonary acute toxicity included a pulmonary embolism in 2 patients requiring hospitalization. Regarding late toxicities, the patients complained most about urogenital side effects, followed by gastrointestinal and musculoskeletal complaints. No toxic effects of grades 4 and 5 were seen.

**Table 2:** Distribution of acute and late toxicities among organ systems and their severity

Affected organ system	Grade 1	Grade 2	Grade 3	Grade 4	Grade 5	Total occurrence
*Acute toxicity*	–					35 (100%)
Gastrointestinal	2 (29%)	5 (71%)	0	0	0	7 (20%)
Hematologic	0	8 (67%)	4 (33%)	0	0	12 (34%)
Musculoskeletal	2 (100%)	0	0	0	0	2 (6%)
Renal/urogenital	4 (44%)	5 (56%)	0	0	0	9 (25%)
Pulmonary	0	0	2 (100%)	0	0	2 (6%)
Cutaneous	0	1 (100%)	0	0	0	1 (3%)
Pain	0	1 (100%)	0	0	0	1 (3%)
Constitutional	0	1 (100%)	0	0	0	1 (3%)
*Late toxicity*	–					14 (100%)
Gastrointestinal	3 (75%)	1 (25%)	0	0	0	4 (29%)
Musculoskeletal	0	1 (100%)	0	0	0	1 (7%)
Urogenital	6 (86%)	1 (13%)	0	0	0	7 (50%)
Constitutional	0	1 (100%)	0	0	0	1 (7%)
Lymphatic	0	1 (100%)	0	0	0	1 (7%)

**Table 3:** Prognostic factors for overall survival

Variables	Overall survival^a^ (%)	*p*-value	DM-free survival^a^ (%)	*p*-value	PR-free survival^a^ (%)	*p*-value
All patients (*n* = 46)	53	–	54	–	83	–
IIB–IIIA (*n* = 24) IIIB–IVA (*n* = 22)	79	0.015*	76	0.012*	89	0.53
	33	–	30	–	74	–
50–65 d (*n* = 28) > 65 d (*n* = 18)	64	0.004*	68	0.008*	84	0.72
	38	–	23	–	83	–
≤ 73Gy (*n* = 19) > 73Gy (*n* = 27)	50	0.046*	36	0.024*	92	0.32
	58	–	69	–	78	–

*DM* distant metastasis, *PR *pelvic recurrence, *OTT *overall treatment time, *D*_*2cc*_* rectum *dose received at rectum at D = 2 cm^3^

^a^5‑year survival rate was calculated

*difference between two groups = statistically significant.

### Survival data and prognostic factors

**Fig. 2:**
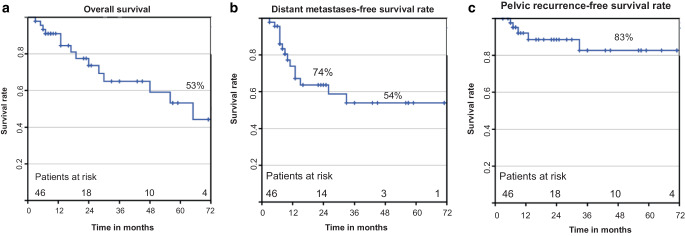
**a** Overall survival, **b** distant metastases-free survival, **c** pelvic tumor-free survival

**Fig. 3:**
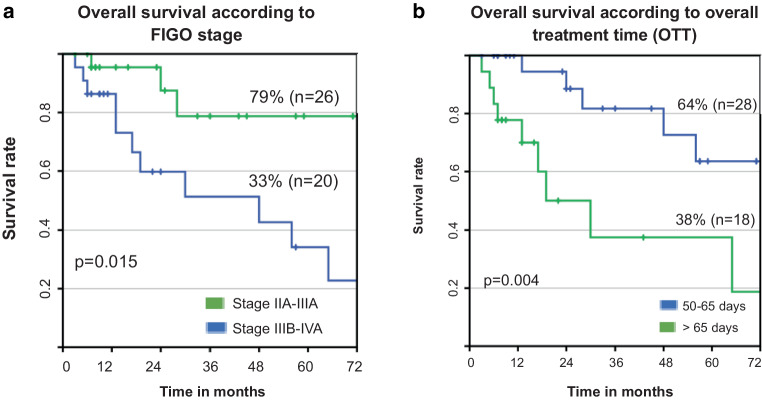
**a** Impact of FIGO stage (IIA–IIIA: 79% vs. IIIB–IVA: 33%, *p* = 0.015), **b** impact of overall treatment time (OTT; 50–65 d: 64% vs. > 65 d: 38%, *p* = 0.004)

Five-year overall survival (OS), distant metastases-free survival (DMFS), and pelvic tumor-free survival rates (PTFS) were 53%, 54%, and 83%, respectively (Fig. 2a–c). A significant impact on OS was seen for FIGO stage (IIA–IIIA: 79% vs. IIIB–IVA: 33%, *p* = 0.015), for overall treatment time (OTT; 50–65 d: 64% vs. > 65 d: 38%, *p* = 0.004), and for rectal D_2cc_ (≤ 73 Gy: 50% vs. > 73 Gy: 38%, *p* = 0.046). Table 3 depicts the prognostic factors with impact on the OS. Figure 3a–c depict the respective survival curves. The identical parameters were significantly associated with DMFS (FIGO stage: *p* = 0.012, OTT: *p* = 0.008, D_2cc_: *p* = 0.024). No parameters with a significant influence on PTFS were seen. In multivariate analysis, an impact of FIGO stage on OS (*p* = 0.05) and DMFS (*p* = 0.014) was detected, and of rectal D_2cc_ on DMFS (*p* = 0.031). Median follow-up time for surviving patients was 21 months (range 3–89 months). No impact on OS, DMFS, and PRFS was noted for histopathologic typing (adenocarcinoma vs. squamous cell carcinoma), patients age at diagnosis, total RT dose, cumulative cisplatin dose, and D_2cc_ of the urinary bladder.

**Fig. 4:**
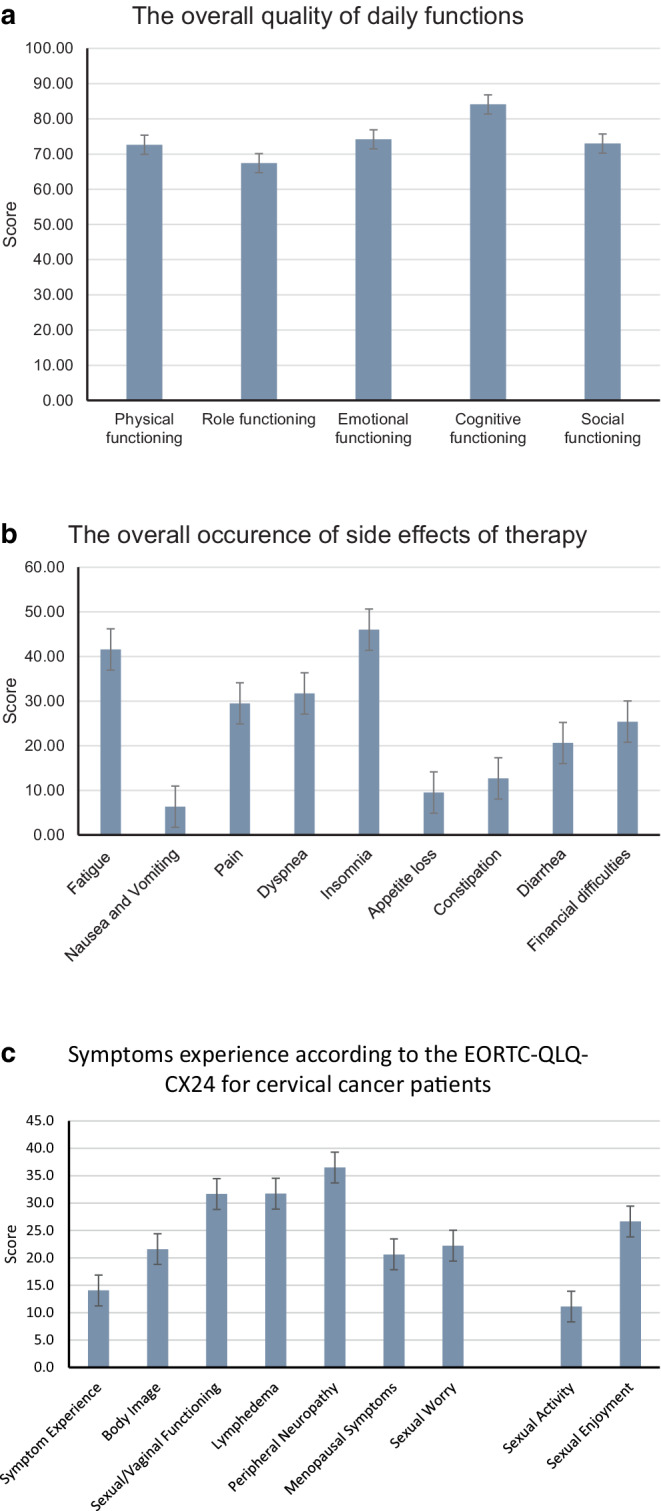
**a** Overall quality of daily functions according to EORTC-QLQ-C30 (data are presented as mean ± standard deviation), **b** overall occurrence of side effects of therapy according to EORTC-QLQ-C30 (data are presented as mean ± standard deviation), **c** functional scale and symptom scale of EORTC-QLQ-CX24 (data are presented as mean ± standard deviation)

### Evaluation of the EORTC-QLQ-C30 and EORTC-QLQ-CX24 questionnaires

Global health score (overall health), general QoL score, and the cervical cancer-specific QoL score after receiving RCT and interstitial BT were assessed. The results are given on a scale of 0 to 100, where 100 is considered full functionality for the functional scales and considered with the worst outcome in symptom scales, whereas 0 is the worst possible outcome for functional scales and the best for the symptom scales. Figure 4a-c depict the QoL-parameters. The outcomes are seen in the tables below (Tables 4 and 5).

**Table 4:** General QoL scores according to the EORTC-QLQ-C30 questionnaire

Scales for QLQ-C30	Mean	IQR	95% CI	Standard deviation
*Global health*				
Global health/QoL	63	50.0–66.6	56.9–70	±14.3
*Functional scales*				
Physical functioning	73	66.6–86.3	65.4–79.9	±15.8
Role functioning	67	58.3–100	55.1–79.8	±27.1
Emotional functioning	74	58.3–91.6	65.7–82.7	±18.6
Cognitive functioning	84	66.6–100	75.6–92.6	±18.6
Social functioning	73	50.0–100	60.2–85.8	±28.1
*Symptom scales*				
Fatigue	41	20.0–66.6	29.5–53.6	±26.5
Nausea and vomiting	6	0–16.6	1.3–11.4	±11.1
Pain	29	16.6–33.3	20.9–38.1	±18.8
Dyspnea	32	0.0–66.6	17.7–45.7	±30.7
Insomnia	46	0–66.6	29.0–62.9	±37.2
Appetite loss	10	0–16.5	1.0–18.0	±18.7
Constipation	13	0–33.3	2.5–22.8	±22.3
Diarrhea	21	0–33.3	6.6–34.6	±30.7
Financial difficulties	25	0–33.3	10.3–40.5	±33.2

*QLQ-C30 *quality of life questionnaire C30, *IQR *interquartile range, *CI *confidence interval, *QoL* quality of life

**Table 5:** Cervical cancer specific parameters according to EORTC-QLQ-CX24 questionnaire

Scales for QLQ-CX24	Mean	IQR	95% CI	Standard deviation
*Symptom scales*				
Symptom experience	14	6.0–16.5	0.8–35.3	±11.9
Body image	22	0–38.3	−21.7–60.5	±29.8
Sexual/vaginal functioning	32	8.3–54.1	−2.9–66.3	±27.9
Lymphedema	32	0–66.6	−25.2–91.8	±32.4
Peripheral neuropathy	36	0–66.6	−18.7–71.9	±34.8
Menopausal symptoms	21	0–33.3	−13.9–93.9	±28.8
Sexual worry	22	0–33.3	5.4–74.5	±30.2
*Functional scales*				
Sexual activity	11	0–16.5	9.6–83.7	±24.3
Sexual enjoyment	27	0–49.9	−7.9–61.2	±27.9

*QLQ-CX24 *quality of life questionnaire CX24, *IQR *interquartile range, *CI *confidence interval

According to the evaluation of the EORTC-QLQ-C30 questionnaire, general health scored an average of 63 out of 100 (IQR: 50.0–66.6; SD ± 14.3). The least impaired was cognitive function, with a score of 84 (IQR: 66.6–100%; SD ± 18.6). Emotional functions had a mean score of 74 (IQR: 58.3–91; SD ± 18.6). The symptom limiting the patients most was insomnia, scoring 46/100 (IQR: 0–66.6%; SD ± 37.2). Thereafter, the most incisive symptoms perceived were fatigue with a mean score of 41 (IQR: 20–66.6; SD ± 26.5), dyspnea with a mean of 32 (IQR: 0–66.6; SD ± 30.7), and pain with an average score of 29 (IQR: 16.6–33.3; SD ± 18.8). Patients complained less frequently about diarrhea, constipation, and loss of appetite.

The last part of the questionnaire could be answered if one had been sexually active in the last 4 weeks. This was true for 5 female patients and showed the result that less than one third of these 5 had enjoyed sex.

## Discussion

For the treatment of patients with locally advanced cervical cancer, cisplatin-based radiochemotherapy followed by brachytherapy is today considered the standard of care [6]. Brachytherapy based on MRI-data after proper tumor shrinkage allows for dose escalation to achieve high tumor control rates and has significantly contributed to avoiding treatment-related complications [7]. In this context, the issue of patient-reported QoL data is becoming increasingly important [1]. Here, using the EORTC-QLQ-C30 and CX24 questionnaires, patient-reported outcomes addressing specific quality of life items after therapy were evaluated to discuss the possible long-term impact of radiochemotherapy and brachytherapy.

As major prerequisite for this evaluation, however, a reasonably high survival rate in this patient group is of utmost importance. In a recent study conducted by Chopra et al., a 5-year survival rate between 53 and 55% was observed after radiochemotherapy followed by brachytherapy [8], which compares nicely with our data. Survival results may be higher in studies that also included patients with an earlier stage of cervical cancer. Here, survival rates of 75 and 76% at 5 years were reported [7, 9]. However, 5‑year survival rates have increased significantly in locally advanced cervical cancer since the 1990s. This is due to the simultaneous administration of chemotherapy and additional interstitial brachytherapy as compared to EBRT alone [8, 10]. FIGO stage was the only independent prognostic factor for OS and DMFS in our series. This was also discussed as a significant prognostic factor in other studies [8, 11–13]. Their results are consistent with those of the present study, in which the 5‑year survival rate for patients with FIGO stage IIB/IIIA was 79% vs. 33% for patients with more advanced disease.

As survival of cancer patients increases, QoL after treatment becomes increasingly important [14]. Analysis of EORTC questionnaires from 21 female patients showed moderately good overall QoL with only slightly reduced functional activities, where all still had a function of at least 67 out of 100. Similar results for overall QoL after radiochemotherapy were reported from a *BMC Women’s Health* study [15]. The functional scale gave the best results for cognitive abilities in our study. Physical, social, and emotional functioning did about equally well, while role functioning did the worst. In other studies, emotional functioning showed the greatest cut, while they reported good physical functioning [16]. The latter is also true for the study presented here. Emotional function is expected to be low, due to patients’ fear of cancer recurrence [15]. In this study of 21 patients who were able to complete the QLQ, only one suffered a recurrence of the disease, which might explain the good overall emotional functioning observed in this patient group. Psychosocial factors could be partly responsible for the low score in role functioning. It has been described that after treatment, women feel less able to continue their role in society, for example as a housewife or a mother [16].

After evaluating the symptom scale of EORTC-QLQ-C30, it was found that the most distressing symptoms after cancer treatment were insomnia and fatigue, followed by dyspnea, pain, and diarrhea. The results are consistent with the most common symptoms reported in studies from India and from Brazil [15, 17]. But in contrast to the aforementioned study, constipation was a less frequently reported problem among the patients of this study. Financial difficulties were in the upper middle range of distressing symptoms. According to the literature, financial difficulties are most stressful immediately after radiotherapy [14]. This is also reflected in this study, as financial difficulties were rated with the highest score by patients who received radiotherapy up to 8 months ago.

Evaluation of the EORTC-QLQ-CX24 revealed that patients most frequently experienced peripheral neuropathy, lymphedema, and sexual/vaginal dysfunction. The worsening of peripheral neuropathy symptoms was also displayed in a study from Brazil [18]. Due to radiation-induced late effects to the vaginal mucosa including atrophy and stenosis of the vagina, the vaginal functions are more frequently impaired [19, 20], as was also observed in other studies [1, 15].

The occurrence of peripheral neuropathy after treatment was also described in another study [15], but the prevalence of lymphedema was lower. Overall, only 5 of the 21 patients had been sexually active within the previous 4 weeks, and one third of them were able to enjoy it. The rare participation in this question, and thus the low sexual activity among the patients, can be partly attributed to the negatively affected body image and the resulting low self-esteem, and partly due to the side effects of the therapy like vaginal dryness, pain, constitutional symptoms, and others. On the other hand, the decrease in sexual activity may be related to decreased sexual activity with increasing age and the increased likelihood of being a widow and thus not having a sexual partner. Similar rates related to sexual enjoyment were also reflected in the study from India [15].

This study is limited by its small sample size. There were 46 eligible patients in total, who received radiochemotherapy and brachytherapy. Of those, 21 answered the EORTC-QLQ-C30 and CX24 questionnaires. In addition, all patients were recruited from one single cancer center. Therefore, the results can only be applied to a larger population to a limited extent. In addition, QoL was only assessed after treatment and also at irregular intervals, so that for each patient, a different amount of time had elapsed between the end of treatment and completion of the questionnaires. In addition, it can be assumed that the more severe the complications of the treatment, the lower the willingness to participate in a study. The willingness to participate in a study is also greater among younger patients.

## Conclusion

In patients with advanced cervical cancer, a combination of RCT and BT remains an excellent treatment option. In terms of patient-reported long-term quality of life, specific support is needed to alleviate symptoms including lymphedema, peripheral neuropathy, and impaired sexual activity.
